# A Fast and Easy Method to Co-extract DNA and RNA from an Environmental Microbial Sample

**DOI:** 10.1264/jsme2.ME22102

**Published:** 2023-03-15

**Authors:** Yusuke Okazaki, Tuyen Thi Nguyen, Arisa Nishihara, Hisashi Endo, Hiroyuki Ogata, Shin-ichi Nakano, Hideyuki Tamaki

**Affiliations:** 1 Institute for Chemical Research, Kyoto University, Gokasho, Uji, Kyoto, 611–0011, Japan; 2 Bioproduction Research Institute, National Institute of Advanced Industrial Science and Technology, Central 6, Higashi 1–1–1, Tsukuba, Ibaraki 305–8566, Japan; 3 Center for Ecological Research, Kyoto University, 2–509–3 Hirano, Otsu, Shiga, 520–2113, Japan

**Keywords:** methodology, DNA extraction, RNA extraction, long-read metagenome, metatranscriptome

## Abstract

We herein propose a fast and easy DNA and RNA co-extraction method for environmental microbial samples. It combines bead beating and phenol-chloroform phase separation followed by the separation and purification of DNA and RNA using the Qiagen AllPrep DNA/RNA mini kit. With a handling time of ~3 h, our method simultaneously extracted high-quality DNA (peak size >10–15‍ ‍kb) and RNA (RNA integrity number >6) from lake bacterioplankton filtered samples. The method is also applicable to low-biomass samples (expected DNA or RNA yield <50‍ ‍ng) and eukaryotic microbial samples, providing an easy option for more versatile eco-genomic applications.

The cultivation-independent, high-throughput sequencing of DNA and RNA has become a standard approach to studying the ecology and evolution of environmental microbes. With the propagation of this method, researchers are examining samples with less accessibility or a lower microbial density, such as the deep waters of the ocean and lake and ultra-oligotrophic groundwaters (Nunoura *et al.*, 2015; Salazar *et al.*, 2016; Okazaki *et al.*, 2017; Nobu *et al.*, 2023). The application of sequencing to these environmental samples is often limited due to the low yield of DNA and RNA, even after tens or hundreds of liters of sample filtration. RNA collection from these environmental samples is challenging because of severe time constraints due to the short half-life of RNA (Steiner *et al.*, 2019). Therefore, the efficient use of DNA and RNA from environmental microbial samples is an important issue in microbial ecology.

While the extraction of DNA and RNA is generally performed separately, the co-extraction of both nucleic acids from the same sample is desirable when the amount of the‍ ‍available sample is limited. Even when the amount of‍ ‍a‍ ‍sample is not a limitation, co-extraction is preferable in‍ ‍cases in which DNA- and RNA-based results will be compared to each other because it will reduce the bias introduced by differences in DNA and RNA extraction procedures (McCarthy *et al.*, 2015). Furthermore, co-extraction is cheaper and faster than the individual extraction of DNA and RNA. In previous studies, DNA and RNA were co-extracted from aquatic, soil, and sediment microbial samples using a combination of bead beating and phenol-chloroform phase separation, followed by the isolation and purification of DNA and RNA with the AllPrep DNA/RNA mini kit (Cat. 80204; Qiagen) (DeAngelis *et al.*, 2010; Gillies *et al.*, 2015; Engelbrektson *et al.*, 2018). Our colleagues recently developed a modified version of this protocol, from which the initial ammonium aluminum sulfate and cetyltrimethylammonium bromide (CTAB) treatment and polyethylene glycol (PEG) precipitation steps were removed (Okazaki *et al.*, 2022). This simplified method minimized the risk of the loss and decomposition of nucleic acids and successfully extracted high-quality DNA and RNA compatible with long- and short-read metagenomes (Okazaki *et al.*, 2022) and metatranscriptomes (Okazaki *et al.*, unpublished) from freshwater microbial samples. In the present study, we examined the methodology in more detail and described our best practice to co-extract DNA and RNA from environmental microbial samples. We optimized the protocol by testing different bead beating conditions and pre- and post-treatment options to demonstrate their effects on the yield, integrity, and purity of extracted nucleic acids.

To obtain environmental samples for methodological development, we collected 30 L of water from a depth of 5‍ ‍m at a pelagic site in Lake Biwa (35°13′09.5″N, 135°59′44.7″E) on October 6th, 2021. The water sample was sequentially filtered through a 200-μm mesh, 5-μm polycarbonate filter (Cat. TMTP14250; Merck Millipore), and Sterivex cartridge with a pore size of 0.22‍ ‍μm (Cat. SVGP01050; Merck Millipore) using a peristaltic pump system. The Sterivex cartridge was replaced for every 1 L of‍ ‍filtration to obtain 30 replicates from a 30-L sample. The 5-μm filter collected all 30 L of the sample and was radially cut into eight equal pieces using a sterilized scalpel. The cartridges and filters were flash-frozen in a dry ice-ethanol bath and stored at –80°C for further processing.

The extraction performance of each experimental treatment was assessed for half of the filter paper removed from the Sterivex cartridge using a sterilized scalpel. We developed an optimum extraction protocol using the prokaryotic fraction (Sterivex filter) and then validated its performance using the eukaryotic fraction (the 5-μm filter). To test the performance of the protocol for low-biomass samples, half of the Sterivex filter was further cut into smaller pieces (cut sizes=1/8, 1/16, 1/32, and 1/64 of half of the Sterivex filter) using a sterilized scalpel and used for extraction. The Qubit 4 fluorometer (Thermo Fisher Scientific), DS-11 spectrophotometer (Denovix), and TapeStation 4150 (Agilent Technologies) were used to assess the quantity, purity (260/280 and 260/230‍ ‍nm absorption ratios), and quality (size distribution) of the extracted nucleic acids, respectively. The output of TapeStation for DNA and RNA was evaluated by the peak size and RNA Integrity Number (RIN) (Schroeder *et al.*, 2006), respectively.

DNA contamination in an RNA sample was tested by qPCR using the prokaryotic universal primers 515F-Y and 926R (Parada *et al.*, 2016). Each of the 10-μL reactions contained 200 nM of each primer, 0.1‍ ‍μL of the RNA extract, and 5‍ ‍μL of KAPA SYBR FAST qPCR Master Mix (2×) (Cat. KK4620, Kapa Biosystems). qPCR was performed using the QuantStudio-1 real-time PCR system (Thermo Fisher Scientific) with initial denaturation at 95°C for 20‍ ‍s, followed by 40 cycles of amplification (denaturation at 95°C for 15‍ ‍s, annealing at 55°C for 20‍ ‍s, and extension at 72°C for 1 s). Two technical replicates were run, and the average of the threshold cycle (Ct) was measured for each sample. In each qPCR plate, three negative controls (the sample was replaced with MilliQ water) were run, and their average Ct was evaluated. Ct of the negative controls ranged between 34 and 35. Melting curve and gel-electrophoresis ana­lyses suggested that non-specific and specific amplification contributed to detection within the negative controls. Specific amplification was difficult to control, presumably due to the extreme universality of the primers and unremovable contaminants from the chemicals. Therefore, we used the difference in Ct between a sample and the negative control (ΔCt) to evaluate the level of DNA contamination in an RNA sample.

Fig. 1 shows the experimental procedure of the proposed method. The essence of this protocol is to reduce the time, cost, and risk of contamination and sample degradation by simplifying the procedure prior to loading a sample onto the spin column of the AllPrep DNA/RNA mini kit. In our experience, the total processing time for 6–8 samples was approximately 3‍ ‍h (without the additional TURBO DNase treatment). The combination of bead beating and phenol-chloroform phase separation in the first step allows quick and effective microbial cell lysis and the removal of insoluble and hydrophobic contaminants. Notably, intrinsic nucleases are instantly inactivated by β-mercaptoethanol and guanidine thiocyanate in the buffer and removed into the phenol phase, thereby minimizing the risk of DNA and RNA decomposition. Regarding sample collection, we recommend the use of a phenol-soluble filter material, such as polyethersulfone (PES) and polycarbonate (PC). This allows the filter to completely dissolve into phenol, which facilitates contact between cells and beads and the separation of the aqueous and phenol layers. When an insoluble filter material, such as polytetrafluoroethylene (PTFE) or polyvinylidene difluoride (PVDF), is used, the filter needs to be chopped into small pieces using a sterilized scalpel before bead beating to allow contact between the beads and cells.

In our previous study (Okazaki *et al.*, 2022), bead beating conditions were set as 3,500‍ ‍rpm for 30‍ ‍s, followed by cooling on ice for 1‍ ‍min, and then at 3,500‍ ‍rpm for 30‍ ‍s again using the MS-100 bead beater (TOMY Digital Biology). After this study, we noted that weaker bead beating conditions extracted less fragmented DNA with a satisfactory extraction efficiency, as described later. To establish optimum conditions, we herein examined different bead beating frequencies (1,800–3,000‍ ‍rpm) and durations (10–30 s) using the μT-12 bead beater (TAITEC). It is important to note that the same beating performance may not be reproducible in different bead beating devices even at the same frequency and duration setting because the beating condition also depends on the rotor’s motion pattern (orbit and amplitude).

We initially tested different beating frequencies by fixing the beating duration at 30‍ ‍s (Fig. 2). The results obtained indicated that a higher frequency resulted in a greater DNA yield (Spearman’s rho=0.81; *P*<0.0001) and shorter DNA peak size (Spearman’s rho=–0.87; *P*<0.0001). The effects of the beating frequency on the RNA yield were unclear due to the large variation among replicates. RIN was always >6, irrespective of the treatments. Based on these results, we selected 2,500‍ ‍rpm as the best condition that balances quality and quantity for general applications (*e.g.*, short-read metagenome, metatranscriptome, and amplicon sequencing) and 1,800‍ ‍rpm as the condition to prioritize the DNA length over the yield for a long-read sequencing ana­lysis. We then investigated the effects of different beating durations by fixing the beating speed at 1,800 and 2,500‍ ‍rpm (Fig. 3). The results obtained indicated that at the same bead beating frequency, the DNA yield was consistently lower at a duration of 10‍ ‍s than at the longer durations, while the effects of the duration on the RNA yield were unclear. DNA and RNA yields and the DNA peak size did not significantly differ between 20 and 30 s. RIN was again always >6, irrespective of the beating duration. Based on these results, we concluded that 20 and 30‍ ‍s of beating satisfied the demand. Although we did not test stronger conditions with a longer duration of beating, previous studies demonstrated that these conditions significantly compromised the integrity of DNA and RNA (Leite *et al.*, 2012; Albertsen *et al.*, 2015).

In the present study, the DNA yield from half of the Sterivex filter was 1.45–1.88‍ ‍μg for the general protocol (2,500‍ ‍rpm for 30 s) and 0.79–1.07‍ ‍μg for the long-DNA protocol (1,800‍ ‍rpm for 30 s) (Supplementary Table S1), which equaled 2.90–3.76 and 1.40–2.14‍ ‍μg of DNA L^–1^ of lake water, respectively. The extraction of microgram-scale DNA from 1 L of lake water was similar to the highest recoveries reported in other oligo-mesotrophic aquatic systems, in which the typical bacterial cell density was in the order of 10^8^ to 10^9^ cells L^–1^ (McCarthy *et al.*, 2015). Therefore, the extraction efficiency of our protocol satisfied the general requirements. However, cells may not have been completely lysed under our relaxed bead beating conditions. Longer or stronger bead beating needs to be considered when yield is a priority, but will compromise the integrity of DNA and RNA, as described above and later.

We assessed the performance of our protocol for low-biomass samples using the same filter replicates cut into smaller pieces. The results obtained indicated that DNA and RNA were both recovered from low-biomass samples from which the expected yield (estimated from the filter cut size and the yield from the uncut filter) was less than 50‍ ‍ng (Supplementary Table S2). The estimated recovery efficiency (measured/expected yield) of DNA exceeded 100% in all cut samples. Values >100% may be attributable to a higher extraction efficiency in smaller filters due to increased cell-bead and cell-chemical contacts. These results suggest that in the uncut filter treatments, cells were not completely lysed or the input biomass or filter amount exceeded the capacity of the bead beating tube. Therefore, splitting samples into different tubes in the bead beating step needs to be considered in order to increase the yield. On the other hand, the recovery efficiency of RNA showed lower values and did not reach 100% in smaller cut sizes (Supplementary Table S2). The loss of RNA in lower biomass samples may be attributable to the incomplete binding and elution of a low amount of RNA during the column purification steps. In summary, the protocol may also co-extract DNA and RNA from low-biomass samples, but may have lower RNA extraction efficiency.

In the previous study, the peak size of extracted DNA was 7–8‍ ‍kb, and the N50 of the resulting long-read (Nanopore) metagenomic sequence reads was 4–5‍ ‍kb (Okazaki *et al.*, 2022), which is consistent with other long-read metagenomic projects (Bertrand *et al.*, 2019; Moss *et al.*, 2020; Trigodet *et al.*, 2022). By relaxing bead beating conditions, we herein achieved a large improvement in the DNA peak size, with an average of >10‍ ‍kb for the general protocol (2,500‍ ‍rpm) and >15‍ ‍kb for the long-DNA protocol (1,800‍ ‍rpm) (Fig. 2). The selection of high-mole­cular DNA using AMPure magnetic beads (Beckman Coulter) or the Short Read Eliminator kit (Pacific Biosciences) may further facilitate obtaining longer sequence reads. It is important to note that our bead beater did not provide a weaker (<1,800‍ ‍rpm) beating condition that may extract even longer DNA. Our method has potential for high-mole­cular-weight DNA extraction from environmental samples, which is a critical issue in the growing field of long-read eco-genomics.

In all bead beating treatments, RIN was always >6 (Fig. 2 and 3), satisfying RNA integrity requirements for general downstream applications. To remove DNA contamination in the RNA sample, we opted for an on-column DNase treatment using the RNase-Free DNase Set (Cat. 79254; Qiagen) as recommended by the manufacturer. The resulting ΔCt ranged between –0.2 and 4.0 (Supplementary Fig. S1), indicating successful, but not complete, DNA removal from the RNA sample, given that ΔCt was 16.1 for the RNA extract without any DNase treatment. To further remove DNA contaminants, we propose an additional DNase treatment using the TURBO DNA-free Kit (Cat. AM1907, Thermo Fisher Scientific) after the final elution of RNA from the spin column (Fig. 1). This additional treatment lowered ΔCt to <2 in all samples, indicating the further removal of DNA contaminants (Supplementary Fig. S1). Our method is effective for isolating high-quality RNA from environmental microbial samples together with DNA, which allows an RNA-based application (*e.g.*, metatranscriptome) to a hard-to-collect environmental sample, for which a DNA-based application is generally prioritized.

We measured the purity of extracted DNA and RNA using a spectrophotometer. A pure nucleic acid extract typically shows 260/280 ratios of ~1.8 and ~2.0 for DNA and RNA, respectively, and 260/230 ratios of 1.8–2.2 for both nucleic acids (Desjardins and Conklin, 2010). However, the samples extracted by our method did not reach these quality standards (Supplementary Fig. S2). Since residual phenol is‍ ‍a common source of a perturbated spectrum, we tested protocols without phenol (*i.e.*, bead beating was performed with RLT buffer only) and with an additional chloroform washing step. However, spectral results were not markedly improved, and DNA and RNA yields were significantly lower in the no-phenol treatment (Supplementary Fig. S2). Therefore, we concluded that the phenol treatment was necessary and not the reason for the compromised spectrum. We presumed that residual guanidinium thiocyanate carried over from the RLT buffer was one of the sources of contaminants. Although guanidinium thiocyanate compromises the absorption spectrum even at concentrations <1‍ ‍mM, no significant effects on Nanopore DNA sequencing (https://community.nanoporetech.com/contaminants) and downstream RNA applications (von Ahlfen and Schlumpberger, 2010) have been reported at concentrations up to 50‍ ‍mM. The previous study (Okazaki *et al.*, 2022) performed short- and long-read metagenomic sequencing of extracted DNA without issues. Purification using AMPure magnetic beads (Beckman Coulter) is recommended when satisfactory 260/280 and 260/230 ratios are desired (data not shown); however, there will be a loss of nucleic acids during the purification step.

We applied the established method to the eukaryotic fraction (5–200‍ ‍μm) filter and successfully extracted high-quality DNA and RNA (Supplementary Table S1). Therefore, our method was effective for both eukaryotic and prokaryotic lake microbial samples. To further confirm the potential of this method, testing with other types of microbial samples, including isolates and non-filter (non-aquatic) samples, is essential. It currently remains unclear whether our simplified method removes impurities inhibiting downstream applications (*e.g.*, PCR and sequencing library preparation) from more contaminant-rich samples (*e.g.*, soil, sediment, and biofilm). When co-extraction is unnecessary (*i.e.*, DNA or RNA is required), a protocol specifically developed to extract DNA or RNA from the target organism or material also needs to be considered. Nonetheless, given its speed and simplicity, our method appears to be one of the primary options when attempting nucleic acid extraction from a novel microbial sample.

In comparisons with methods based on chemical or enzymatic lysis, those based on mechanical lysis by bead beating are known to generate a less biased result in terms of the microbial community composition (Pollock *et al.*, 2018). However, the extent of the bias was not investigated in the present study because we did not sequence our samples. Since a compositional bias may be observed even among different bead beating conditions (Albertsen *et al.*, 2015), fixed beating conditions need to be applied when comparing community compositions among multiple samples.

In conclusion, our method provides a fast and easy option to co-extract DNA and RNA from environmental microbial samples and will enable more versatile DNA- and RNA-based applications, including long-read metagenomics and metatranscriptomics.

## Citation

Okazaki, Y., Nguyen, T. T., Nishihara, A., Endo, H., Ogata, H., Nakano, S., and Tamaki, H. (2023) A Fast and Easy Method to Co-extract DNA and RNA from an Environmental Microbial Sample. *Microbes Environ ***38**: ME22102.

https://doi.org/10.1264/jsme2.ME22102

## Supplementary Material

Supplementary Material

## Figures and Tables

**Fig. 1.**
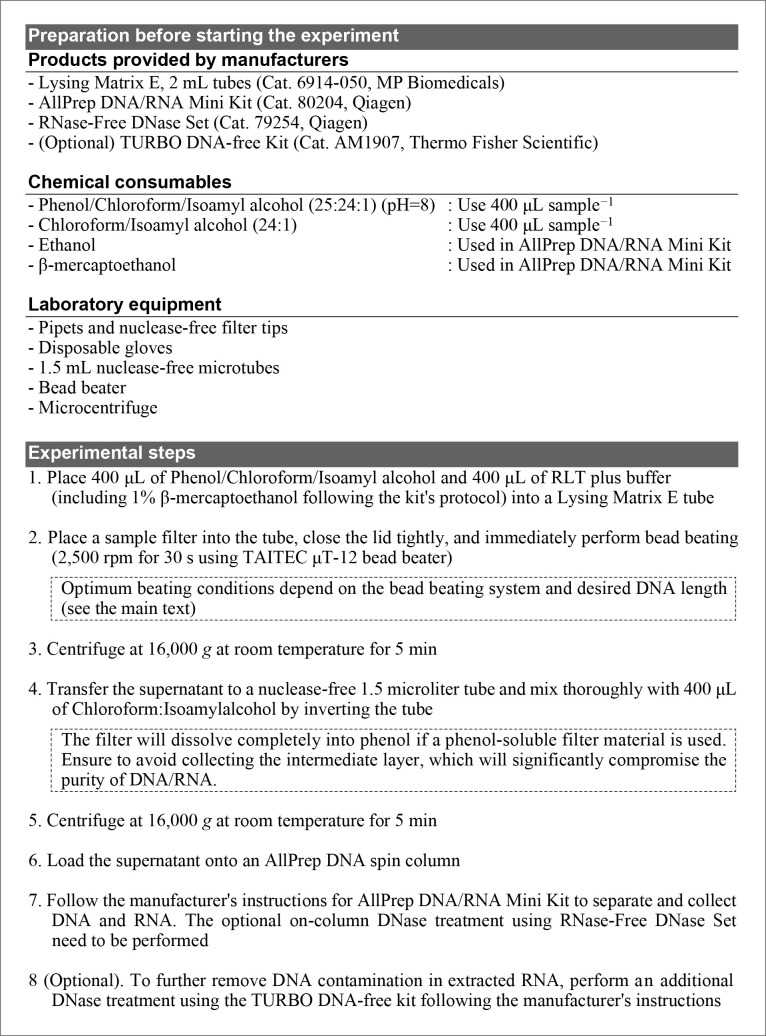
Experimental procedure of the method proposed in the present study.

**Fig. 2.**
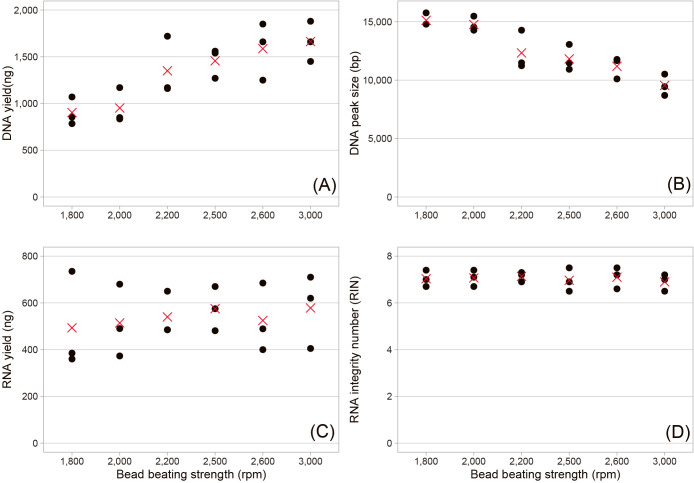
Effects of different bead beating strengths on (A) DNA yield, (B) DNA peak size, (C) RNA yield, and (D) RNA integrity number (RIN). The black points indicate the results of each triplicate, and the red crosses indicate their average. Raw data are available in Supplementary Table S1.

**Fig. 3.**
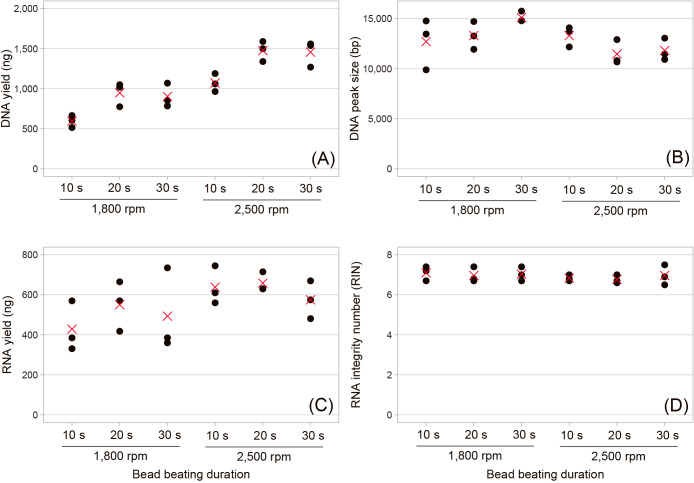
Effects of different bead beating durations at beating strengths of 1,800 and 2,500‍ ‍rpm on (A) DNA yield, (B) DNA peak size, (C) RNA yield, and (D) RNA integrity number (RIN). The black points indicate the results of each triplicate, and the red crosses indicate their average. Raw data are available in Supplementary Table S1.
