# Supplementary material for: A Fast and Easy Method to Co-extract DNA and RNA from an Environmental Microbial Sample
Source: Microbes Environ. 2023 Mar 15;38(1):ME22102. doi: 10.1264/jsme2.ME22102 (PMC10037101; doi:10.1264/jsme2.ME22102)
Supplement: Supplementary file 1 — Supplementary Material [file 38_22102_s1.pdf]

Supplementary Information for:

A Fast and Easy Method to Co-extract DNA and RNA from an Environmental Microbial Sample

Yusuke Okazaki, Tuyen Thi Nguyen, Arisa Nishihara, Hisashi Endo, Hiroyuki Ogata,
Shin-ichi Nakano, Hideyuki Tamaki

Corresponding author:
Yusuke Okazaki
Email: okazaki.yusuke.e31@kyoto-u.jp

This PDF file includes:

Supplementary Figures S1 and S2

Supplementary Tables S1 and S2

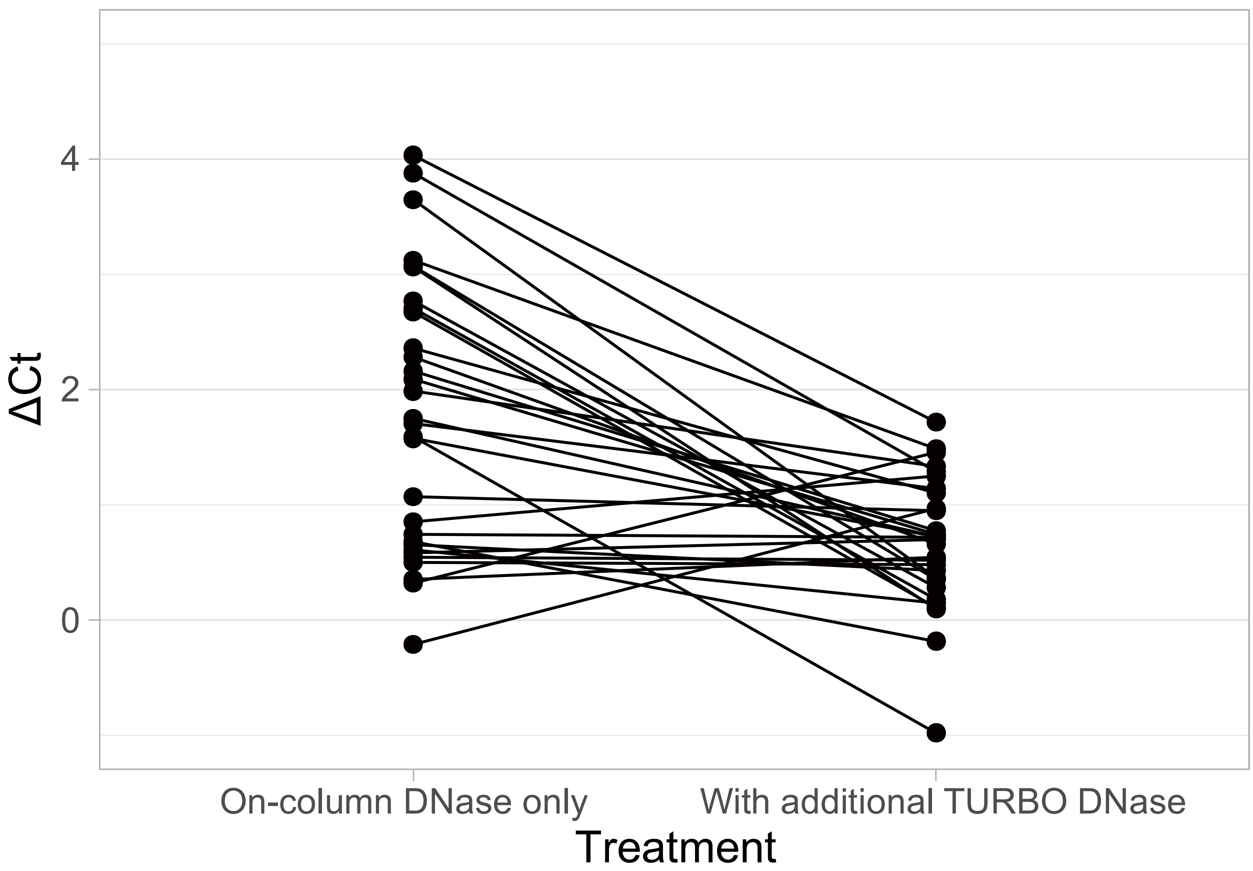

Supplementary Figure S1. Effect of the additional TURBO DNase treatment on removing DNA contamination in the extracted RNA. ΔCt was measured for each of the 30 extracted RNA samples (shown in Figures 2 and 3) before (i.e., only with the on-column DNase treatment using RNase-Free DNase Set) and after the additional TURBO DNase treatment. The result from the same sample was linked by a line to demonstrate the shift of ΔCt before and after the treatment. Raw data is available in Supplementary Table S1.

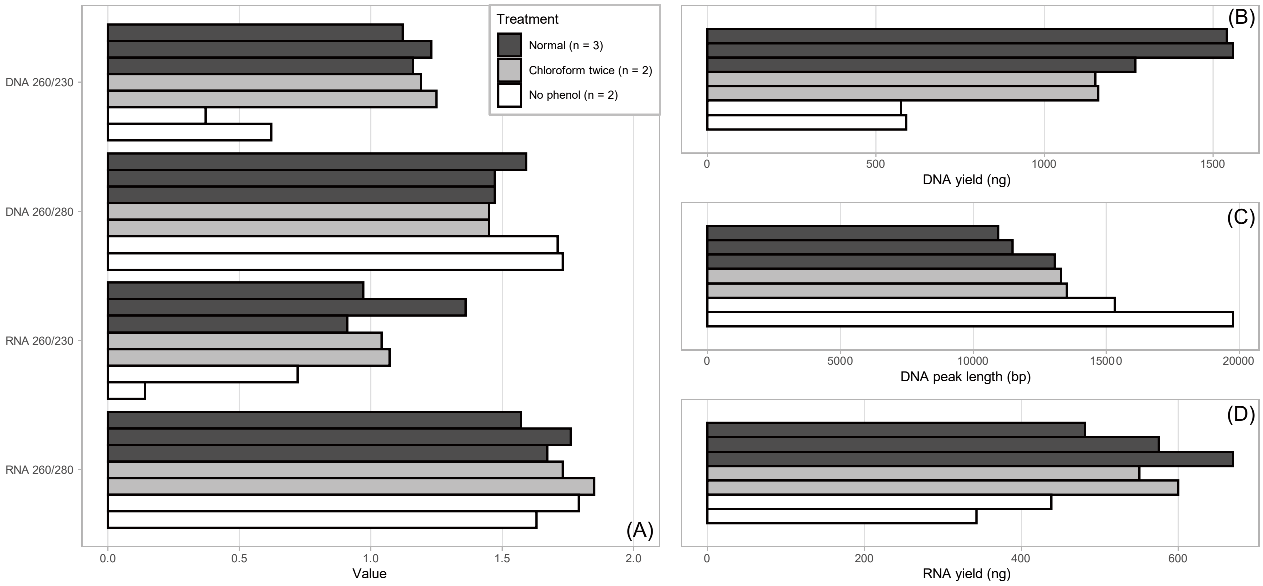

Supplementary Figure S2. Effect of additional chloroform washing step and removal of phenol in the initial bead beating step on (A) DNA and RNA purity measured by a spectrophotometer, (B) DNA yield, (C) DNA peak length, and (D) RNA yield. The bead beating condition was fixed to 2500 rpm for 30 s in this comparison. Raw data is available in Supplementary Table S1.

				DNA				RNA						
Beating frequency (rpm)	Beating Duration (s)	Specific Treatment	Replicate	DNA yield (ng)	260/230	260/280	Peak size (bp)	RNA yield (ng)	260/230	260/280	RIN	ΔCt On-column Dnase only	ΔCt With additional TURBO DNase	RNA/DNA ratio
1800	30		1	785	1.07	1.51	14795	360	1.51	1.47	7.4	0.609	0.151	0.46
2000	30		1	850	0.96	1.47	14535	373	1.27	1.67	7.4	1.985	1.331	0.44
2200	30		1	1170	0.5	1.39	11235	485	0.43	1.58	7.3	3.069	0.356	0.41
2500	30		1	1540	1.12	1.59	10930	481	0.97	1.57	7.5	0.852	1.248	0.31
2600	30		1	1850	1.15	1.59	10106	489	1.07	1.59	7.5	2.362	1.106	0.26
3000	30		1	1880	1.08	1.54	8698	710	1.56	1.46	7.2	3.880	1.289	0.38
1800	30		2	1070	1.15	1.43	15758	735	1.34	1.76	6.7	0.683	-0.182	0.69
2000	30		2	1170	1.03	1.43	15475	680	1.4	1.8	6.7	0.351	0.544	0.58
2200	30		2	1720	1.23	1.45	11485	650	0.87	1.65	6.9	1.073	0.949	0.38
2500	30		2	1560	1.23	1.47	11467	575	1.36	1.76	6.5	2.285	0.663	0.37
2600	30		2	1660	1.3	1.48	11673	685	0.9	1.83	6.6	2.706	0.181	0.41
3000	30		2	1450	1.09	1.45	10513	620	1.48	1.94	6.5	2.088	0.744	0.43
1800	30		3	855	1.23	1.38	14794	385	0.65	1.65	7	-0.209	0.970	0.45
2000	30		3	835	0.82	1.27	14274	490	1.11	1.62	7.1	0.655	0.434	0.59
2200	30		3	1160	1.12	1.29	14274	485	1.1	1.6	7.2	0.585	0.701	0.42
2500	30		3	1270	1.16	1.47	13057	670	0.91	1.67	6.9	1.575	0.727	0.53
2600	30		3	1250	1.15	1.41	11774	400	0.76	1.66	7.2	2.158	0.774	0.32
3000	30		3	1660	1.18	1.51	9437	405	1	1.83	7	0.746	0.717	0.24
1800	10		1	515	0.97	1.35	9891	331	0.88	1.36	7.4	0.321	1.456	0.64
1800	20		1	775	1.24	1.34	11940	418	0.34	1.36	7.4	2.673	0.110	0.54
2500	10		1	965	1.16	1.44	14089	745	1.09	6	6.7	3.067	0.098	0.77
2500	20		1	1340	1.14	1.45	12910	715	0.5	1.75	6.7	4.035	1.720	0.53
1800	10		2	665	1.1	1.4	13466	570	1.49	1.93	6.7	1.708	1.142	0.86
1800	20		2	1050	1.13	1.44	14720	665	1.32	1.79	6.7	3.649	0.367	0.63
2500	10		2	1060	1.23	1.45	13676	610	1.35	1.8	6.8	2.767	0.282	0.58
2500	20		2	1590	1.28	1.49	10681	630	1.12	1.73	6.6	3.120	1.489	0.40
1800	10		3	605	1.06	1.37	14773	385	1.56	1.58	7.2	0.502	0.482	0.64
1800	20		3	1020	0.96	1.45	13273	570	0.81	1.59	6.8	0.548	0.521	0.56
2500	10		3	1190	1.05	1.48	12178	560	0.64	1.59	7	1.592	-0.977	0.47
2500	20		3	1500	0.69	1.49	10811	630	0.61	1.56	7	1.751	0.727	0.42
2500	30	Chloroform wash twice	1	1150	1.19	1.45	13301	550	1.04	1.73	7	2.105		0.48
2500	30	Chloroform wash twice	2	1160	1.25	1.45	13505	600	1.07	1.85	7.1	1.897		0.52
2500	30	Without phenol	1	575	0.37	1.71	15318	438	0.72	1.79	7.4	4.258		0.76
2500	30	Without phenol	2	590	0.62	1.73	19775	343	0.14	1.63	7.3	2.476		0.58
2500	30	Eukaryotic filter sample	1	1060	1.2	1.44	14087	970	0.51	2.02	5.8	2.413		0.92
2500	30	Eukaryotic filter sample	2	1170	0.94	1.47	15201	790	1.08	2.28	6.2	1.480		0.68

Supplementary Table S1. The raw data of the comparative extraction experiments.

Average DNA yield from an uncut filter (ng)	1455	[A]
Average RNA yield from an uncut filter (ng)	575	[B]

		DNA			RNA		
Filter cut size [C]	Replicate	Expected yield (ng) [A*C]	Actual yield (ng)	Actual/Expected	Expected yield (ng) [B*C]	Actual yield (ng)	Actual/Expected
1/8	1	182	434	239%	72	104	145%
1/8	2	182	426	234%	72	99	138%
1/16	1	91	138.5	152%	36	31.57	88%
1/16	2	91	129	142%	36	21.53	60%
1/32	1	45	71	156%	18	ND (<10)	<56%*
1/32	2	45	51	112%	18	ND (<10)	<56%*
1/64	1	23	28.2	124%	9	ND (<10)	-
1/64	2	23	23.2	102%	9	ND (<10)	-

ND, not detected (The detection limit for RNA quantification was 10 ng in this study)

*The maximum recovery efficiency was estimated from the detection limit for RNA qualtification (10 ng)

Supplementary Table S2. The raw data of the low-biomass sample extraction experiments.
